# Emerging Presence of Culturable Microorganisms in Clinical Samples of the Genitourinary System: Systematic Review and Experience in Specialized Care of a Regional Hospital

**DOI:** 10.3390/jcm11051348

**Published:** 2022-03-01

**Authors:** Antonio Rosales-Castillo, Gemma Jiménez-Guerra, Lara Ruiz-Gómez, Manuela Expósito-Ruíz, José María Navarro-Marí, José Gutiérrez-Fernández

**Affiliations:** 1 Servicio de Medicina Interna, Virgen de las Nieves University Hospital & Instituto de Investigación Biosanitaria de Granada (ibs.GRANADA), Avenida de las Fuerzas Armadas 2, 18014 Granada, Spain; anrocas90@hotmail.com; 2 Program in Clinical Medicine and Public Health, University of Granada & Instituto de Investigación Biosanitaria de Granada (ibs.GRANADA), 18016 Granada, Spain; 3 Laboratory of Microbiology, Virgen de las Nieves University Hospital & Instituto de Investigación Biosanitaria de Granada (ibs.GRANADA), Avenida de las Fuerzas Armadas 2, 18014 Granada, Spain; bobdiegjg@gmail.com (G.J.-G.); josem.navarro.sspa@juntadeandalucia.es (J.M.N.-M.); 4 Department of Microbiology, School of Medicine, University of Granada & Instituto de Investigación Biosanitaria de Granada (ibs.GRANADA), Avenida de la Investigación 11, 18016 Granada, Spain; lararuizgomez@correo.ugr.es; 5 Unit of Biostatistics, Department of Statistics, School of Medicine, University of Granada, 18016 Granada, Spain; mexpositoruiz@ugr.es

**Keywords:** urinary tract infection, emerging pathogens, cervix bacteria, vaginitis bacteria, balano-posthitis bacteria, prostatitis bacteria, epididymitis bacteria, urethritis bacteria

## Abstract

The detection of emerging pathogens responsible for genitourinary infections has increased with technological advances. We conducted a systematic review of publications on the involvement of these microorganisms in genitourinary samples, and we also investigated their presence and antibiotic susceptibility in samples from patients at our regional hospital (Granada, Spain). The MEDLINE database was searched up to 31 December 2020, and a cross-sectional descriptive study was performed of results obtained in urine samples and genital exudates from January 2016 through December 2019. The review highlighted the frequent involvement of *Neisseria meningitidis* in genital infections, while the data on other microorganisms were consistent with findings in our patient series. The emerging microorganisms most often responsible for urinary tract infections were *Streptococcus bovis* (58.5%) and *Gardnerella vaginalis* (23.6%) in females, and *S. bovis* (32.3%), *Aerococcus urinae* (18.6%), and *Corynebacterium* spp. (16.9%) in males; those most frequently reported in genital infections were *S. viridans* (36.4%) in females and *C. glucuronolyticum* (32.2%) and *G. vaginalis* (35.6%) in males. In general, emerging pathogens are resistant to conventional antibiotics such as penicillin. However, there has also been an increase in beta-lactam resistance by the *S. bovis* group and *Corynebacterium* spp. The systematic review showed that emerging microorganisms are responsible for only a small percentage of genitourinary infections but are of major clinical interest, with a predominance of the *S. bovis* group, *G. vaginalis*, *Lactobacillus* spp., *Aerococcus* spp., and *Corynebacterium* spp. in urine samples and of *G. vaginalis* and *C. glucuronolyticum* in genital samples. Given the increasing resistance to antibiotics empirically prescribed in patients with genitourinary infections, it is recommended to create an antibiogram in all cases.

## 1. Introduction

Genitourinary infections are among the most frequent infections worldwide and are responsible for a large percentage of hospital and primary care consultations, and a delay in their correct diagnosis and treatment can sometimes have serious consequences for patients. The microbiological agents most widely considered in diagnostic protocols are *Enterobacteriaceae*, *enterococci*, *Pseudomonas* spp., *Acinetobacter* spp., and *Candida* spp. However, major technological advances now permit the detection of microbiological agents that had not previously been identified as responsible for infections and whose cultures had tested negative, resulting in the prescription of non-targeted broad spectrum empirical antibiotics.

The capacity to identify these less well-known agents allows an antibiogram to be created, which is of major clinical relevance because of their resistance to the antibiotics most often empirically prescribed in this type of infection (e.g., quinolones and fosfomycin). This allows the selection of a targeted antibiotic treatment, with the associated microbiological and ecological advantages. Early descriptions in the literature of infrequent microorganisms involved in cases of cystitis and prostatitis [1,2,3,4] have been followed by an increasing number of reports as microbiological techniques and culture methods have improved. There are various possible explanations for the failure to consider these microorganisms, including their misclassification due to the absence of distinctive phenotypic criteria or the misinterpretation of significant growth as “contamination by microbiota”, and their non-detection by standard methods due to their slow growth and the need for nutritionally enriched culture media [5]. They include *Corynebacterium* spp., *Aerococcus* spp., *Actinotignum* spp., *Lactobacillus* spp., *Streptococcus bovis* (SBG) and *viridans* (SVG) groups, *Facklamia* spp., *Pasteurella* spp., *Neisseria meningitidis*, and *Gardnerella vaginalis*. Their development and emergence in genitourinary tract infections have been favored by multiple social factors, including the early initiation of sexual relations by adolescents, among other societal trends. They are of particular concern because they can arise in the absence of the pathogens habitually associated with genitourinary tract infections and are increasingly frequent in older populations with more concomitant diseases. Given the lack of information in the literature on these microorganisms and the increase in their detection through improved clinical microbiology techniques, studies are needed to update diagnostic and therapeutic protocols accordingly. Hence, the objective of this study was to analyze the presence of infrequent microorganisms in genitourinary samples through a systematic review of the literature and a study of the microorganisms detected in our regional hospital and their antibiotic susceptibility. 

## 2. Material and Methods

### 2.1. Systematic Review 

The MEDLINE database was searched for relevant publications up to 31 December 2020. The terms “*urinary tract infection*” and “*emergent pathogens*” were used in an open search and the terms *cervix bacteria*, *vaginitis bacteria*, *balanitis bacteria*, *posthitis bacteria*, *prostatitis bacteria*, *epididymitis bacteria*, and *urethritis bacteria* in an open search filtered by “Case Report” and “Letter”. Review exclusion criteria were: studies on microorganisms habitually involved in genitourinary infections (*Enterobacteriaceae*, glucose non-fermenting gram-negative bacilli, *Candida* spp., or *Hemophilus* spp.); studies on *Staphylococcus* spp. And some *Streptococcus* spp., including only SBG, SVG, and *Streptococcus pneumoniae*; studies on microorganisms responsible for sexually transmitted infections; studies that did not report on the relationship of microorganisms with a clinical situation, and studies that were not published in English or Spanish. The references of all studies were reviewed to complete the search.

### 2.2. Study of Suspected Clinical Episodes

A cross-sectional descriptive study was performed on microbiology laboratory results obtained in samples from patients suspected of genitourinary system infection attended between 1 January 2016 and 31 December 2019 by specialist, emergency, and outpatient care departments of the Virgen de las Nieves University Hospital in Granada (Spain), a regional specialist center serving over 330,000 inhabitants. No exclusion criteria were applied, except for the exclusion of duplicates and repeat microbiological studies of the same episode. 

Urine samples from patients suspected of urinary tract infection (UTI) were gathered from mid-stream micturition, permanent catheterization, provisional catheterization, pediatric urine collection bag, or nephrostomy catheter under anti-contamination conditions and were processed as previously described, using a 1-μL calibrated loop and UriSelect 4 chromogenic culture medium (Bio-Rad, Barcelona, Spain) incubated for 24 h at 37 °C. In samples from the Nephrology Department alone, a lamb blood agar plate (Becton-Dickinson, Madrid, Spain) was added and incubated in CO_2_. Cutoff points for colony growth were: Negative (<10,000 UFC/mL and <1000 UFC/mL in urine from provisional catheterization); Positive (bacteriuria >100,000 UFC/mL of one or two uropathogens, or between 10,000 and 100,000 of one alone; and >10,000 UFC/mL of one or two uropathogens, or between 1000 and 10,000 UFC/mL of one alone in urine from provisional catheterization); or Mixed (>10,000 UFC/mL of more than two uropathogens).

Semen, glans, endocervical, genital ulcer and urethral exudate samples from patients suspected of genital system infection were processed as previously described, using real-time multiplex PCR to investigate the presence of *C. trachomatis*, *N. gonorrhoeae*, *T. vaginalis* (BD MAX CT/GC/TV BD, Franklin Lakes, NJ, USA), *Mycoplasma genitalium*, *Mycoplasma hominis*, and *Ureaplasma urealyticum* (BD MAX System, BioGX DNA, 350-011-A-MAX, Amsterdam, The Netherlands). In samples of vulvovaginal exudates, DNA hybridization tests were used to detect *G. vaginalis*, *Candida* spp., and *trichomonas* (BD AFFIRM VPIII, BD, Madrid, Spain) in a BD MicroProbe Processor, and they were also seeded on blood agar (Becton-Dickinson), chocolate agar (Becton-Dickinson), Martin–Lewis agar (Becton-Dickinson) media for *N. gonorrhoeae* and on Chromogenic agar medium for *Candida* spp. (BIO-RAD). Results were considered significant when there was monomicrobial and abundant growth (up to the third seeding area) of an opportunistic pathogen or the presence of a strict pathogen. The presence of *M. hominis* and *U. urealyticum* was indicated when the study yielded a value of Ct ≤ 30. More information on the sensitivity of the PCR system to detect mycoplasmas and ureaplasmas is available from the manufacturer (https://drive.google.com/drive/folders/1KuQwlMsrmBlQx_3ftQHc4T3re1ZdO_tk (accessed on 30 November 2021)). Given that a value of Ct ≤ 30 indicates a higher microorganism concentration and M. hominis and *Ureaplasma* spp. Can be potential colonizers, these were only reported in samples with this result.

Optimal identification of the isolated microorganisms was performed using MALDI-TOF mass spectrometry (Biotyper, Brucker Daltonics, Billerica, MA, USA) and/or MicroScan Walkaway (Beckman-Coulter, Brea, CA, USA). The susceptibility study was conducted by broth microdilution using the automated MicroScan Walkaway system for SBG and diffusion gradient strips (MIC Test Strip, Liofilchem, Italy) for the remaining microorganisms, as recommended by the European Committee on Antimicrobial Susceptibility Testing (EUCAST) (https://eucast.org/ (accessed on 30 November 2021)) [6] and/or Clinical and Laboratory Standards Institute (CLSI) (https://clsi.org/ (accessed on 30 November 2021)) [7] for the year of isolation. In the absence of reference data, use was made of interpretation criteria for microorganisms that are similar in respiratory metabolism, Gram staining behavior, and growth rate. Isolated microorganisms were classified in the laboratory reports as susceptible, intermediate, or resistant to tested antibiotics, accompanied by a request for clinical assessment of the report.

Data were gathered from the Andalusian public health laboratory computer system (MODULAB ^®^) on type of sample, origin, microorganism, and patient sex and age for their anonymized evaluation. The microorganisms excluded from the literature search (see above) were also excluded from this study (Enterobacteriaceae, glucose non-fermenting gram-negative bacilli, *Candida* spp., *Hemophilus* spp., *Staphylococcus* spp., selected *Streptococcus* spp., and microorganisms producing sexually transmitted infections). Episodes in which the microorganism of interest was isolated alongside another microorganism were also excluded, given the impossibility of distinguishing the one responsible for the associated disease. The objectives were to evaluate the results obtained for monomicrobial culturable microorganisms with an abundant and significant presence and to determine the rate of emerging pathogens in genitourinary system infections at our hospital from 2016 through 2019. In a descriptive analysis, absolute and relative frequencies were calculated for each type of sample, grouped by microorganism. The relationship between the presence of microorganisms and age or sex was evaluated using Pearson’s chi-square test or Fisher’s test (when no more than 20% of cells had expected frequencies <5), considering *p* < 0.05 as significant. Finally, the susceptibility of microorganisms to antibiotics was calculated by clinical category. IBM SPSS Statistics 19 was used for data analyses. Adequate clinical information was not available to analyze factors associated with the presence of a given microorganism.

## 3. Results 

### 3.1. Systematic Review

The search of the literature yielded 54 items on emerging microorganisms in genitourinary samples (see Table 1, Table 2 and Table 3 and Table S1). The most frequently reported UTI-producing microorganisms were *Corynebacterium* spp., *Aerococcus* spp., and *Actinotignum* spp. (Table 1) The most frequently reported urethritis-producing microorganism in males were *Neisseria meningitides* (15 cases), *Corynebacterium* spp., and *Streptococcus* spp. (Table 2). In genital episodes other than urethritis, the most frequent were *N. meningitidis* and *Actinotignum schaalii*, with three cases each (Table 3). 

In the non-filtered search, 950 articles were initially retrieved for “*urinary tract infection”* and “*emerging pathogens*”, and 12 of these met eligibility criteria for inclusion (Table 1); 699 articles were retrieved for *urethritis bacteria*, of which 26 were included in the review (Table 2), 580 for *cervix bacteria*, of which three were included; 1574 for *vaginitis bacteria*, of which 4 were included (Table 3); 79 for “*balanitis or posthitis” bacteria*, of which one was included; and 620 for “*prostatitis or epididymitis*” *bacteria*, of which six were included (Table 3).

### 3.2. Study of Suspected Clinical Episodes

From January 2016 through December 2019, 50,823 clinical episodes were microbiologically studied for suspicion of UTI, and 15,736 (30.96%) were positive for significant bacteriuria, including 223 (1.4%) produced by culturable emerging microorganisms. Among 2618 episodes with suspicion of genital infection, 1.113 (42.5%) were positive, including 88 (7.9%) produced by emerging microorganisms (Table 4 and Table 5). Over the same period, 45 positive genitourinary episodes were recorded in 2016, 80 in 2017, 109 in 2018, and 77 in 2019. In samples from urinary episodes, the most frequent emerging microorganisms were SBG (91 episodes), followed by *Gardnerella vaginalis*, *Aerococcus urinae*, *Aerococcus sanguinicola*, and, in nephrology patients and patients aged >65 years, *Lactobacillus* spp. 

By sex (Table S2), the most frequent emerging microorganisms in UTI episodes were SBG (58.5%) and *G. vaginalis* (23.6%) in females and *Aerococcus* spp. (28.8%) and *Corynebacterium* spp. (16.9%) in males (*p* < 0.001). By age (Table S3), the most frequent microorganism was SBG (88.9%) in children (aged ≤ 14 years), whereas there was a wider distribution of microorganisms in adults (aged > 14 years): SBG in 45.7%, *G. vaginalis* in 23.2%, *Aerococcus* spp. in 15.9%, *Lactobacillus* spp. in 7.9%, and *Corynebacterium* spp. in 7.3%) (*p* < 0.016). By sex (Table S4), the most frequent emerging microorganisms in episodes of genital infection were *G. vaginalis* (35.6%) and *Corynebacterium glucuronolyticum* (32.2%) in males (all adults), and SVG (45.5%) and *Eikenella corrodens* (27.3%) in females (almost all adults) (*p* < 0.001).

Urethral samples were all from male patients, and the most frequent microorganisms were *G. vaginalis* (*n* = 10 isolates) and *C. glucuronolyticum* (*n* = 5). In endocervical exudates (*n* = 24), the most frequent microorganism was SVG (*n* = 7). In semen and glans exudate samples, the most frequent microorganisms were *C. glucuronolyticum* (*n* = 13) and *G. vaginalis* (*n* = 11). 

The susceptibility of the detected uropathogens is exhibited in Table 6, highlighting the resistances obtained for *Corynebacterium* spp., SBG, SVG and *Lactobacillus* spp. Among genital infections (Table 7, Table 8 and Table 9), resistance to clindamycin and metronidazole was observed for *Actinobaculum massiliensis* and *Actinotignum schaalii* and resistance to some penicillins for *Neisseria meningitidis*. 

## 4. Discussion

Bacterial infections of the genitourinary tract are frequently empirically treated with generic antibiotics because routine laboratory procedures are unable to provide a diagnosis. However, advances in the diagnostic procedures available to clinical microbiology laboratories (e.g., mass spectrometry—MALDI-TOF, media enrichment and molecular techniques) have improved identification of the microorganisms responsible for these infections, allowing specifically targeted treatments to be applied in a larger proportion of cases [8].

Overall, the findings of the systematic review of 54 selected studies are comparable with the results obtained in the laboratory of our hospital. They indicate that microorga-nisms responsible for genital infection can sometimes be opportunistic and may or may not be usually present in the genital tract. For instance, they can be introduced into the genital tract during sexual activity without barrier protection or from intrauterine devices, tampons, and exploratory medical procedures, among others, and pregnancy and previous disease may also play a role. Hence, understanding of the development of disease by emerging microorganisms is hampered by the influence of numerous and heterogeneous predisposing factors [9]. The evidence presented here (Table 2 and Table 3) confirms the implication of these microorganisms in episodes of genital infection. In line with findings published in the reviewed articles, the microorganisms most frequently detected among males in the present series were *N. meningitidis*, *Corynebacterium* spp., streptococci and, finally, *G. vaginalis*. It has been reported that *G. vaginalis* forms part of the urogenital microbiota in 7–11% of males and may represent a focus of infection [10,11]. Likewise, the predominant microorganisms in females were *S. anginosus* and *S. constellatus*, which form part of the oral, respiratory, and gastrointestinal microbiota but can produce clinical infections when outside their usual habitat [12]. The low percentage susceptibility of these infections to penicillin and levofloxacin, frequently associated with resistance to macrolides, cotrimoxazole, tetracyclines, streptogramin B, or lincosamides [13], hinders their treatment. Three isolates of *E. corrodens* were detected in samples from females; this microorganism is part of the oral microbiota but can produce gynecological infections, frequently polymicrobial, including pelvic inflammatory disease in IUD carriers and chorioamnionitis [14]. In contrast, genus *Pasteurella* spp. is infrequent in genital samples and its detection is therefore of particular interest [15]. *Facklamia hominis* is also a highly infrequent pathogen in humans, although its prevalence may be underestimated, and it was detected in samples from episodes of UTI, urethritis, and balanoposthitis in the present study. The variability of its susceptibility profile complicates the management of infection with this microorganism, and there is no established empirical treatment [16,17], so that antibiotic susceptibility studies are necessary. In the present series, isolates of *Moraxella osloensis* were detected in samples from episodes of urethritis and genital infection. This microorganism is susceptible to most of the studied antibiotics except for azithromycin, although the most appropriate therapy is not well established [18,19,20]. The isolate of *Alloscardovia omnicolens* detected was not susceptible to metronidazole or moxifloxacin, as previously reported [21,22]. *Leptotrichia* can be part of the oral microbiota, and immunosuppression or vaginal mucosal erosion can be predisposing factors for disease after translocation, and it is usually resistant to moxifloxacin [23,24]. Finally, *N. meningitidis* has been isolated in the ge-nital tract as a cause of urethritis and is commonly susceptible to most studied antibiotics, including amoxicillin, ceftriaxone, and doxycycline [25], although resistance to penicillin and ampicillin was observed in the present study.

In relation to UTI, both the reviewed articles and the present study describe the frequent presence of *Corynebacterium* spp., *Aerococcus* spp. and *Actinotignum* spp. which have been considered contaminants, possibly favoring their underdiagnosis. Moreover, some of these have phenotypic characteristics similar to those of enterococci or streptococci, as in the case of *Aerococcus* spp., leading to frequent confusion. A further cause of the underestimation of *Corynebacterium* spp. as a UTI producer may have been the absence of enriched media to achieve their growth [5], given that they usually grow slowly with minuscule colonies on blood agar medium in the presence of CO_2_. In the present series, blood agar culture medium was used for at-risk populations, including nephrology patients and those of an advanced age, facilitating the detection of emerging microorganisms. Emerging uropathogens include the SBG, especially *Streptococcus gallolyticus*, subspecies *pasteurianus*, although species and subspecies could not be specified in all cases and these data are therefore not reported; however, studies using MALDI-TOF have shown that almost all SBG isolates correspond to the aforementioned subspecies [26]. Most episodes with SBG isolates were in adult women, in line with a previous report that *S. gallolyticus* is a potential agent responsible for bacteriuria in females with a history of urological disease and/or diabetes [12]. The ability to select the appropriate therapy is limited by the lack of clinical cutoff points to interpret antibiograms, which have only been established for ampicillin, penicillin, vancomycin, teicoplanin, and clindamycin. Epidemiological cutoff points are frequently used to overcome this limitation, and in general there have been reports of resistance to erythromycin, clindamycin, levofloxacin, and cotrimoxazole, especially for *S. gallolyticus* [27]. *G. vaginalis* is currently considered a possible uropathogen with likely sexual transmission to males, explaining its increased detection in male urine cultures and genital disease, although it continues to be underdiagnosed [11]. It was detected in urine samples from nephrology patients thanks to the utilization of blood agar medium (see above). Another relevant genus is *Aerococcus* spp., highlighting the predominance of *A. urinae* and *A. sanguinicola* in elderly males with underlying urological disease, who are at higher risk of UTI from infrequent pathogens [28]. Nevertheless, its possible presence should also be considered in adolescents because the diagnosis is often delayed and potentially severe disease (e.g., pyelonephritis, bacteremia, endocarditis or peritonitis) can develop [29]. It is therefore important to rule out the genus *Aerococcus* genus when a urine culture has a significant alpha-hemolytic microorganism count before reporting it as urogenital microbiota. Antibiotic susceptibility cutoff points for *Aerococcus* spp. were published by EUCAST in 2017 [30] and by CLSI in 2015 [31]. This genus is usually susceptible to beta-lactams, the antibiotic of choice, and to vancomycin, which is reserved for allergic patients in combination with gentamycin [32]. However, *A. viridans* has elevated minimum inhibitory concentration (MIC) values for penicillin and aminoglycosides, and resistance to vancomycin has been reported [33,34]. Both the review and our laboratory results evidence elevated MICs for nitrofurantoin, fluoroquinolones, and cotrimoxazole [28]. The susceptibility varies among species, and a susceptibility study is necessary before the selection of antibiotic. *Lactobacillus gasserii* and *Lactobacillus delbueckii* were frequently detected in our urine samples, especially in women of advanced age. Both, especially the former, are considered contaminant microbiota as part of the normal vaginal, gastrointestinal, and oropharyngeal microbiota that can translocate to the urinary system. They have a low virulence, except in patients with immunosuppression or mucosal erosion or undergoing endoscopic procedures [35]. There are documented cases of endocarditis, chorioamnionitis, and renal abscesses in patients with underlying kidney disease, and this possibility should be considered by clinicians [36,37]. All emerging microorganisms under study were mainly detected in adults, and their isolation in samples from children has been exceptional. In fact, the only frequent microorganism observed in the children was SBG, with 16 episodes. 

In our laboratory, *Corynebacterium* spp. and *Actinotignum* spp. were frequently present in urine cultures and genital exudates, highlighting the presence of *C. urealyticum* and *C. glucuronolyticum*, which was especially prevalent among males. Most species of this genus are mucoepithelial microbiota, which can be opportunistic pathogens of the genitourinary system. In cases of suspected infection by *Corynebacterium* spp., incubation should be prolonged when the culture remains negative at 24 h given their slow growth [38], or blood agar media should be used. *Corynebacterium* spp. have undergone the largest number of susceptibility studies because of an increase in the resistance of this genus over recent years. The data from our hospital reveal resistance to penicillin and gentamycin and a high degree of resistance to ciprofloxacin, tetracyclines and lincosamides, limiting the usefulness of these antibiotics [38,39,40]. *A. schaalii* and *A. sanguinis* species of the genus *Actinotignum* were isolated in the present series, and *A. schaalii* was frequently associated with UTI. The presence of this genus may be underdiagnosed because the identification is technically challenging and a prolonged culture time is required, with the need to apply molecular techniques in some cases. Its presence has been related to humidity in the ge-nital area due to diaper use or incontinence, among other causes [41]. *Actinotignum* is commonly resistant to fluoroquinolones and cotrimoxazole, and empirical treatment with beta-lactams is recommended [42]. The most prevalent species in the present study was *A. schaalii*, which is resistant to metronidazole, clindamycin and, occasionally, to nitrofurantoin and gentamicin. It is susceptible to beta-lactams (except for mecillinam), tetracyclines, vancomycin, rifampicin, and linezolid [43,44]. *Actinobacullum massiliensis* is phylogenetically close to *Actinotignum* spp. and may be part of the genitourinary tract microbiota, but it is also a potential pathogen [45].

This is an observational single-center study with no control group. A further potential limitation is that isolates might have translocated from habitats in which they form part of the microbiota (in which case they are of clinical interest) or they might have been part of the microbiota at the site of their detection. However, the presence of isolates was only reported when detected in a non-habitual localization in order to strengthen the scientific rigor of the study. In addition, strict microbiological criteria were applied, including only those with monomicrobial presence and a significant count in urine cultures or an abundant presence in genital exudates. Habitual pathogens were excluded. Special consideration was also given to samples from nephrology and elderly patients. 

Although the data presented here may suggest a change in the bacterial flora causing genitourinary infections, this possibility is not supported by the present study because cultures were not followed over time. It should also be noted that exclusion of the most prevalent causes of infection [46,47] creates an incorrect picture of changes in the flora responsible for genitourinary infections. Nevertheless, further clinical research is needed to evaluate responses to the antibiotic treatments.

## 5. Conclusions

Emerging microorganisms are responsible for a small but clinically relevant proportion of genitourinary infections and are frequently resistant to antibiotics empirically prescribed to treat genitourinary infections, such as ciprofloxacin and fosfomycin. It is essential to consider them among possible genitourinary pathogens and to create an antibiogram when required.

## Figures and Tables

**Table 1:** Articles in the systematic review on emerging microorganisms in urinary tract infections.

Article	Age-Sex	Predisposing Factors	Microorganism	Clinical Manifestations	Clinical Sample	Treatment	Microbiological Identification Method
Vedel G et al., 2006	64-Male	Recurrent urolithiasis	*Corynebacterium pseudogenitalium*	Urinary tract infection	Urine	Norfloxacin	RNAr 16S gene sequencing
El Sayegh H et al., 2007	61-Male	Vesicocutaneous fistula	*Corynebacterium urealyticum*	Cystitis	Urine	Quinolones	Not described
Perciaccante A et al., 2007	57-Male	Systemic lupus erythematosus Left obstructive uropathy with hydronephrosis	*Corynebacterium urealyticum*	Cystitis	Urine	Teicoplanin	Not described
Beteta A et al., 2009	34-Female	Not described	*Corynebacterium striatum*	Urinary tract infection	Urine	Ciprofloxacin	API Coryne (BioMérieux)
Larios OE et al., 2010	76-Female	Recurrent urinary tract infection	*Actinotignum schaalii*	Urinary tract infection	Urine	Clindamycin	Not described
Zimmermann P et al., 2012	8-Male	Neurogenic bladder dysfunction	*Actinotignum schaalii*	Urinary tract infection	Urine	Trimethoprim-Sulfamethoxazo-le Amoxicilin	RNAr 16S gene sequencing
Barberis C et al., 2018	36-Female	Chronic renal failure	*Corynebacterium coyleae*	Urinary tract infection	Urine	Piperacillin/tazo-bactam Ciprofloxacin	MALDI-TOF PCR amplification rpoB gene sequence analysis
Jiménez G et al., 2018	80-Male	Benign prostate hypertrophy	*Aerococcus sanguinicola*	Urinary septic shock Kidney failure	Urine	Amoxicilin-clavulanic acid	MALDI-TOF RNAr 16S gene sequencing
	88-Male	Not described	*Aerococcus sanguinicola*	Urinary tract infection	Urine	Cefuroxime	MALDI-TOF RNAr 16S gene sequencing
Lorenzin G et al., 2018	69-Male	Type 2 diabetes mellitus Terminal renal disease Other comorbidities	*Myroides odoratimimus*	Urinary tract infection	Urine	Trimethoprim-Sulfamethoxazo-le	MALDI-TOF RNAr 16S gene sequencing
Figueroa F et al., 2019	55-Male	Not described	*Aerococcus urinae*	Urinary tract infection Mitral valve endocarditis	Urine Blood	Gentamicin Penicillin G	Not described
Pichon M et al., 2019	67-Female	Neurogenic bladder Recurrent urinary tract infection	*Corynebacterium riegelii*	Urinary sepsis	Urine Blood	Amoxicilin Gentamicin	MALDI-TOF
Napolitani M et al., 2019	20-Male	Suprapubic catheter	*Kocuria kristinae*	Urinary tract infection	Not described	Not described	Not described

**Table 2:** Articles in the systematic review on emerging microorganisms in urethritis.

Article	Age-Sex	Predisposing Factors	Microorganism	Clinical Manifestations	Clinical Sample	Treatment	Microbiological Identification Method
Gregory JE et al., 1979	27-Male	Alcohol abuse	*Neisseria meningitidis*	Urethritis	Urethral exudate	Procaine penicillin Probenecid	Not described
Karolus JJ et al., 1980	29-Male	Oral-genital and vaginal contact	*Neisseria meningitidis*	Urethritis	Urethral exudate	Procaine penicillin	Not described
Chowdhury MNH et al., 1984	35-Male	Sexual relations with female partner (non- extramarital, oral-genital, or anal)	*Streptococcus* group *B*	Urethritis	Urethral exudate	Phenoxymethylpenicillin	Not described
Noble RC et al., 1985	25-Male	Vaginal and oral sexual relation with a female	*Streptococcus pneumoniae*	Urethritis	Urethral exudate	Ampicillin Probenecid	Not described
Hay PE et al., 1989	16-Male	Oral sex (female)	*Neisseria meningitidis*	Urethritis	Urethral exudate	Spectinomycin Doxycycline	Not described
Wilson APR et al., 1989	18-Male	Sexual relations with steady partner (female)	*Neisseria meningitidis*	Urethritis	Urethral exudate	Ampicillin Probenecid	Not described
Phillips EA et al., 1989	19-Male	Sexual contact (female)	*Neisseria meningitidis*	Urethritis	Urethral exudate	Amoxicillin Doxycycline	Not described
Shanmugaratnam K et al., 1989	25-Male	Oral-genital contact	*Neisseria meningitidis*	Urethritis	Urethral exudate	Ciprofloxacin	Not described
Faigel HC et al., 1990	20-Male	Oral-genital contact (female)	*Neisseria meningitidis*	Urethritis	Urethral exudate	Not described	Not described
Coker DM et al., 1991	36-Male	Sporadic sexual relations (female)	*Moraxella urethralis*	Urethritis	Urethral exudate	Ciprofloxacin	Not described
Quarto M et al., 1991	35-Male	Occasional oral sex with a female	*Neisseria meningitidis*	Urethritis	Urethral exudate	Ampicillin	Not described
Kanemitsu N et al., 2003	48-Male	Oral-genital contact	*Neisseria meningitidis*	Urethritis	Urethral exudate	Levofloxacin	Enzymatic profiles
Orden B et al., 2004	36-Male	Unprotected oral and vaginal sexual relations	*Neisseria meningitidis*	Urethritis	Urethral exudate	Ceftriaxone Doxycycline	API NH (bioMérieux)
Rodríguez CN et al., 2005	27-Male	Oral and vaginal sexual relations with several women	*Neisseria meningitidis*	Urethritis	Urethral exudate	Ceftriaxone Doxycycline	API NH (bioMérieux)
Urra E et al., 2005	38-Male	Sexual relations with steady partner (female)	*Neisseria meningitidis*	Urethritis	Urethral exudate	Minocycline	API NH (bioMérieux)
Abdolrasouli A et al., 2007	23-Male	Unprotected fellatio	*Moraxella catarrhalis*	Urethritis	Urethral and throat exudate	Ciprofloxacin	Not described
Koroglu M et al., 2007	43-Male	Multiple sexual partners	*Streptococcus pneumoniae*	Urethritis	Urethral exudate	Amoxicillin	BD BBL Crystal test
Galán-Sánchez F et al., 2011	18-Male	Sexual relations with multiple partners	*Corynebacterium glucuronolyticum*	Urethritis	Urethral exudate	Ciprofloxacin	API Coryne sistem
Katz AR et al., 2011	26-Male	Oral and vaginal sex with one woman	*Neisseria meningitidis*	Urethritis	Urethral exudate	Cefixime Azithromycin	API NH (bioMérieux)
Bousquet A et al., 2012	35-Male	Unprotected oral-genital sex with males and females	*Neisseria meningitidis*	Urethritis	Urethral exudate	Ceftriaxone Azithromycin	MALDI-TOF
Abdolrasouli A et al., 2013	27-Male	Possible unprotected sexual relations	*Corynebacterium propinquum*	Urethritis	Urethral exudate	Azithromycin Vancomycin	API Coryne system (bioMérieux)
Babics A et al., 2015	36-Male	Not described	*Gardnerella vaginalis*	Urethritis	Urine Blood	Azithromycin Ceftriaxone	MALDI-TOF (Bruker)
Gherardi G et al., 2015	37-Male	Not described	*Corynebacterium glucuronolyticum*	Genitourinary tract infection	Urethral exudate Semen Urine	Ciprofloxacin	MALDI-TOF RNAr 16S gene sequencing
Seynabou Lo et al., 2015	52-Male	Previous urethroplasties	*Corynebacterium aurimucosum*	Urinary tract infection	Urine	Imipenem	MALDI-TOF
Grandolfo M et al., 2016	39-Male	Not described	*Neisseria elongata nitroreducens*	Purulent balanoposthitis Urethritis	Urethral exudate	Ceftriaxone Topical mupirocin	Vitek 2 compact system (bioMérieux)
Jannic A et al., 2019	22-Male	Sexual relations with steady partner (female)	*Neisseria meningitidis*	Urethritis	Urethral exudate	Ceftriaxone Azithromycin	MALDI-TOF

**Table 3:** Articles in the systematic review on emerging microorganisms in episodes other than urethritis.

Article	Age-Sex	Predisposing Factors	Microorganism	Clinical Manifestations	Clinical Sample	Treatment	Microbiological Identification Method
**CERVICITIS**							
Jaffe LR et al., 1983	16-Female	Not described	*Neisseria meningitidis*	Pelvic inflammatory disease	Vaginal exudate	Procaine penicillin Probenecid Ampicillin	Not described
Quentin R et al., 1991	80-Female	Genital adenocarcinoma	*Pasteurella multocida*	Metrorrhagia Mucopurulent vaginal secretion Occasional fever	Vaginal exudate	Amoxicillin- clavulanic acid Metronidazole	Not described
Harriau P et al., 1997	19-Female	Pregnancy	*Neisseria meningitidis*	Endocervical infection	Endocervical exudate Urine	Amoxicillin	Not described
**VAGINITIS**							
Greif Z et al., 1986	34-Female	Contact with farm parasites and birds Pregnancy	*Pasteurella multocida*	Septicemia	Blood Vaginal exudate	Cephalotin Tobramycin	Not described
Vila de Muga M et al., 2008	5-Female	Not described	*Streptococcus pneumoniae*	Vaginitis-peritonitis	Vaginal exudate	Ceftriaxone Amoxicillin	Not described
Chen X et al., 2015	9-Female	Not described	*Corynebaacterium amycolatum*	Vaginitis	Vaginal exudate	Topical benzalkonium chloride Amoxicillin	Vitek-2 compact bacterial identification system (bioMérieux) MALDI-Biotyper
Gómez C et al., 2018	28-Female	Not described	*Moraxella osloensis*	Tumor in right groin Right adenopathy	Vaginal exudate	Azithromycin	MALDI-TOF RNAr 16S gene sequencing
**BALANITIS**							
Grandolfo M et al., 2016	39-Male	Not described	*Neisseria elongata nitroreducens*	Purulent balanoposthitis Urethritis	Urethral exudate	Ceftriaxone Topical mupirocin	Vitek 2 compact system (bioMérieux)
**PROSTATI-TIS/EPIDIDY-MITIS**							
Nguyen C et al., 1990	39-Male	Suprapubic removal of vesical calculus	*Streptococcus mutans*	Prostatic abscess	Purulent abscess material	Ampicillin Gentamicin Ceftriaxone Amoxicillin	Not described
QU L et al., 2003	37-Male	Transplantation of part of the intestine (Crohn’s disease)	*Nocardia asteroides*	Prostatitis	Urine	Ciprofloxacin Ampicillin-Sulbactam Ceftriaxone Trimethoprim-Sulfamethoxazole	Not described
Martinaud C et al., 2008	92-Male	Prostatic adenoma Arterial hypertension Parkinson	*Actinotignum schaalii*	Sepsis Prostatitis	Urine	Ofloxacin Ceftriaxone Gentamicin Amoxicillin	RNAr 16S gene sequencing
Torres E et al., 2013	48-Male	Arterial hypertension Left ventricular hypertrophy Renal failure	*Actinotignum schaalii*	Prostatitis	Seminal fluid Urine	Amoxicillin-clavulanic acid	MALDI-TOF RNAr 16S gene sequencing
Siller M et al., 2016	43-Male	Not described	*Actinotignum schaalii*	Chronic prostatitis	Urethral exudate	Amoxicillin-clavulanic acid	MALDI-TOF
Kawahara K et al., 2018	29-Male	Not described	*Neisseria meningitidis*	Prostatitis Arthritis	Urine	Not described	Not described

**Table 4:** Presence of emerging microorganisms in the series of clinical samples.

Microorganism	Urine Male	Urine Female	Endocervical Exudate	Urethral Exudate	Glans Exudate	Semen	Total
*Actinobaculum massiliensis*	2	1	1				4
*Actinotignum schaalii*	2	2	1	1	2	1	9
*Actinotignum sanguinis*					1	1	2
*Actinomyces turicensis*	4		1				5
*Aerococcus christensenii*			1				1
*Aerococcus urinae*	11	7			2	1	21
*Aerococcus sanguinicola*	6	4				1	11
*Aerococcus viridans*		1					1
*Aeromonas hydrophila*			1				1
*Alloscardovia omnicolens*		1	1			1	3
*Corynebacterium amycolatum*		4			1	1	6
*Corynebacterium aurimucosum*	1						1
*Corynebacterium glucuronolyticum*	5	1	1	5	1	12	25
*Corynebacterium jeikeium*	1	1					2
*Corynebacterium minutissimum*		1					1
*Corynebacterium striatum*	2						2
*Corynebacterium urealyticum*	5	1	1				7
*Eikenella corrodens*			3				3
*Facklamia hominis*	1	1	1	1	1	2	7
*Gardnerella vaginalis*	9	29		10	1	10	59
*Lactobacillus crispatus*		3					3
*Lactobacillus delbrueckii*		3					3
*Lactobacillus fermentum*		1					1
*Lactobacillus gasserii*	4	6					10
*Lactobacillus iners*						1	1
*Lactobacillus jensenii*		4					4
*Lactobacillus rhamnosus*	1	1					2
*Leptotrichia trevisanii*			1				1
*Moraxella osloensis*			1	1			2
*Neisseria meningitidis*			2				2
*Pasteurella bettyae*			1	2			3
*Streptococcus* group *bovis*	19	72				3	91
*Streptococcus* group *viridans (anginosus*, *constellatus)*	4	2	7		1		14
*Streptococcus pneumoniae*			1				1
TOTAL	77	146	24	20	10	34	311

**Table 5:** Annualized presence of emerging microorganisms in study of genital infection in males and females.

Microorganism	2016	2017	2018	2019	Total
*Actinotignum schaalii*			3	2	5
*Actinobaculum massiliensis*			1		1
*Actinotignum sanguinis*			1	1	2
*Actinomyces turicensis*		1			1
*Aerococcus christensenii*			1		1
*Aerococcus urinae*			2	1	3
*Aerococcus sanguinicola*				1	1
*Aeromonas hydrophila*	1				1
*Alloscardovia omnicolens*			1	1	2
*C. amycolatum*			2		2
*C. glucuronolyticum*			11	8	19
*C. urealyticum*			1		1
*Eikenella corrodens*		2	1		3
*Facklamia hominis*			3	2	5
*Gardnerella vaginalis*	2	2	7	10	21
*Lactobacillus iners*	1				1
*Leptotrichia trevisanii*		1			1
*Moraxella osloensis*		2			2
*Neisseria meningitidis*	1	1			2
*Pasteurella bettyae*		1		2	3
*Streptococcus* group *bovis*	2	1			3
*Streptococcus* group *viridans (anginosus*, *constellatus)*	1	2	1	4	8
*S. pneumoniae*			1		1
Total	8	13	35	32	89

**Table 6:** Percentage antibiotic susceptibility of emerging microorganisms isolated in UTI *.

Agent		Susceptibility (%)									
		*A. massili-ensis* (*n* = 3)	*A. schaalii* (*n* = 4)	*A. urinae* (*n* = 18)	*A. sanguinicola* (*n* = 10)	*A. viridans* (*n* = 1)	*Corynebacterium* (*n* = 22)	*F. hominis* (*n* = 2)	Lactobaci-llus^**^ (*n* = 23)	*S.* group *bovis* (*n* = 91)	*S.* group *viridans* (*n* = 6)
**Pen**	P	100	100	90	100	0	8		100	90	33
	AMP		100	100	100			0	70	100	50
	AMC	100							100		
	TZP	100									
**Cef**	CTX		100	100	100	100	14	100			100
	CFM						0				
**Carba**	IPM	100	100				0		70		
	MEM	100		90	100		29		100		100
**Quino**	CIP			88	22	100	27				
	LEV		0				0	100	0	77	50
	MXF	33	100								
**Amg**	CN			0			69				
**Gcp**	VA	100	100	100	100					100	100
**Tetra**	TE		100	100			92			21	100
**Ntm**	MTZ	0	0						0		
**Rif**	RD			100	100		100				
**Various**	SXT			0	0						
	FOS						0		0	99	
	F			100	100					100	

* Antibiogram not performed for *A. turicensis* (*n* = 4), *A. omnicolens* (*n* = 1), or *G. vaginalis* (*n* = 38). ** *Lactobacillus*: *crispatus*, *delbrueckii*, *fermentum*, *gasseri*, *jensenii*, *rhamnosus*. Penicillins (Pen), Cephalosporins (Cef), Carbapenems (Carba), Quinolones (Quino), Aminoglycosides (Amg), Glycopeptides (Gcp), Tetracycline (Tetra), Nitroimidazole (Ntm), Rifampicin (Rif), Sulfamides (Sulf), Amoxicillin-Clavulanic (AMC), Ampicillin (AMP), Penicillin (P), Piperacillin-Tazobactam (TZP), Cefotaxime (CTX), Cefixime (CFM), Imipenem (IPM), Meropenem (MEM), Ciprofloxacin (CIP), Levofloxacin (LEV), Moxifloxacin (MXF), Gentamicin (CN), Teicoplanin (TEC), Vancomycin (VA), Tetracycline (TE), Metronidazole (MTZ), Rifampicin (RD), Trimethoprim-Sulfamethoxazole (SXT), Fosfomycin (FOS), Nitrofurantoin (F).

**Table 7:** Percentage antibiotic susceptibility of emerging microorganisms isolated in urethral exudate *.

Agents		Susceptibility (%)				
		*A. schaalii* (*n* = 1)	*C. glucuronolyticum* (*n* = 5)	*F. hominis* (*n* = 1)	*M. osloensis* (*n* = 1)	*P. bettyae* (*n* = 2)
**Pen**	P		20	100		100
	AMP	100			100	100
	AMC			100	100	100
**Cef**	CTX		40		100	
**Carba**	IMP			100		
	MEM		0		100	
**Quino**	CIP		40			100
	LEV				100	
	MXF			100		
**Amg**	CN		60			
**Gcp**	VA	100	100	100		
**Tetra**	TE	100	60			
	DO					100
**Mcr**	E		0			
	AZM				100	
**Linco**	DA	100	0	0		
**Oxa**	LZD		100			
**Ntm**	MTZ	0		0		
**Rif**	RD		100			
**Sulf**	SXT		100		100	100

* Antibiogram was not performed for *G. vaginalis* (*n* = 10). Penicillins (Pen), Cephalosporins (Cef), Carbapenems (Carba), Quinolones (Quino), Aminoglycosides (Amg), Glycopeptides (Gcp), Tetracycline (Tetra), Macrolides (Mcr), Lincosamides (Linco), Oxazolidone (Oxa), Nitroimidazole (Ntm), Rifampicin (Rif), Sulfamides (Sulf), Amoxicillin-Clavulanic (AMC), Ampicillin (AMP), Penicillin (P), Piperacillin-Tazobactam (TZP), Cefotaxime (CTX), Imipenem (IPM), Meropenem (MEM), Ciprofloxacin (CIP), Levofloxacin (LEV), Moxifloxacin (MXF), Gentamicin (CN), Vancomycin (VA), Tetracycline (TE), Doxycycline (DO), Erythromycin (E), Azithromycin (AZM), Clindamycin (DA), Linezolid (LZD), Metronidazole (MTZ), Rifampicin (RD), Trimethoprim-Sulfamethoxazole (SXT).

**Table 8:** Percentage antibiotic susceptibility of emerging microorganisms isolated in genital samples from females *.

Agent		Susceptibility (%)											
		*A. massilien-sis* (*n* = 1)	*A. schaa-lii* (*n* = 1)	*A. hydrophi-la* (*n* = 1)	*A. omnico-lens* (*n* = 1)	*Corynebacte-rium* (*n* = 2)	*E. corro-dens* (*n* = 3)	*L. trevisa-nii* (*n* = 1)	*M. osloensis* (*n* = 1)	*N. meningiti-dis* (*n* = 2)	*P. bettyae* (*n* = 1)	*S. pneumo-niae* (*n* = 1)	*S.* group *viridans* (*n* = 7)
**Pen**	P	100						100		0		100	86
	AMP			0	100		100		100	0	100		
	AMC	100	100	0		100	100	100	100		100	100	
	TZP			100									
**Cef**	KZ			0									
	FOX			100									
	CXM			100									
	CTX			100			100		100	100		100	83
	CFM												
	CAZ			100									
	FEP			100									
**Carb**	ETP			100									
	IMP	100		100			100	100					100
	MEM								100	100		100	100
**Mbac**	ATM			100									
**Quino**	NA			0									
	CIP			0		50	100			100	100	100	
	LEV						100		100	100		100	50
	MXF							0				100	
**Amg**	AK		100	100									
	CN			100		100							
	TOB			100									
**Gcp**	TEC												100
	VA	100	100			100						100	100
**Tetra**	TE					100				100			0
	DO										100		
	TGC			100									
**Mcr**	E					100						0	50
	AZM						33		0	100			0
**Linco**	DA	0	0		100	50		100					83
**Oxa**	LZD					100						100	100
**Ntm**	MTZ	0	0					0					
**Rif**	RD					100							
**Sulf**	SXT			100		0			100	100	100		100

* Antibiogram was not performed for *A. turicensis* (*n* = 1), *A. christensenii* (*n* = 1), or *Facklamia hominis* (*n* = 1). Penicillins (Pen), Cephalosporins (Cef), Carbapenems (Carba), Monobactams (Mbac), Quinolones (Quino), Aminoglycosides (Amg), Glycopeptides (Gcp), Tetracycline (Tetra), Macrolides (Mcr), Lincosamides (Linco), Oxazolidone (Oxa), Nitroimidazole (Ntm), Rifampicin (Rif), Sulfamides (Sulf), Amoxicillin-Clavulanic (AMC), Ampicillin (AMP), Penicillin (P), Piperacillin-Tazobactam (TZP), Cefazolin (KZ), Cefoxitin (FOX), Cefuroxime (CXM), Cefotaxime (CTX), Cefixime (CFM), Ceftazidime (CAZ), Cefepime (FEP), Ertapenem (ETP), Imipenem (IPM), Meropenem (MEM), Aztreonam (ATM), Nalidixic acid (NA), Ciprofloxacin (CIP), Levofloxacin (LEV), Moxifloxacin (MXF), Amikacin (AK), Gentamicin (CN), Tobramycin (TOB), Teicoplanin (TEC), Vancomycin (VA), Tetracycline (TE), Doxycycline (DO), Tigecillin/Tigecycline (TGC), Erythromycin (E), Azithromycin (AZM), Clindamycin (DA), Linezolid (LZD), Metronidazole (MTZ), Rifampicin (RD), Trimethoprim-Sulfamethoxazole (SXT), Daptomycin (DAP), Fosfomycin (FOS), Nitrofurantoin (F).

**Table 9:** Percentage antibiotic susceptibility of emerging microorganisms isolated in semen and glans exudate samples *.

Agent		Susceptibility (%)							
		*A. schaalii* (*n* = 3)	*A. sanguinis* (*n* = 2)	*A. urinae*, *A. sanguinicola* (*n* = 4)	*A. omnicolens* (*n* = 1)	*C. glucuronolyticum* (*n* = 13)	*F. hominis* (*n* = 3)	*L. iners* (*n* = 1)	*S.* group *bovis* (*n* = 3)
**Pen**	P	100	100	100	0	54	100		
	AMP	100		100			100	100	67
	AMC	100			100				
**Cef**	CTX			100		100	100		
**Carba**	IMP	100			100	100		100	
	MEM			100					
**Quino**	CIP			100		23	100		
	LEV			100			100		33
	MXF		100		0				
**Amg**	CN					0			
**Gcp**	VA	100	100	100	100	100	100	100	100
**Tetrac**	TE	100	100	100		46	100		0
**Mcr**	E					77	100		0
	AZM						0		
**Linco**	DA	100			100	23			67
**Oxa**	LZD				100		100		100
**Ntm**	MTZ	0	0		0				
**Rif**	RD			100		100			
**Sulf**	SXT	0				100			
	FOS					0			100
	F			100					

* Antibiogram was not performed for *C. amycolatum* (*n* = 2), *G. vaginalis* (*n* = 11), or *S. viridans* group (S. *anginosus*) (*n* = 1). Penicillins (Pen), Cephalosporins (Cef, Carbapenems (Carba), Quinolones (Quino), Aminoglycosides (Amg), Glycopeptides (Gcp), Tetracycline (Tetra), Macrolides (Mcr), Lincosamides (Linco), Oxazolidone (Oxa), Nitroimidazole (Ntm), Rifampicin (Rif), Sulfamides (Sulf), Amoxicillin-Clavulanic (AMC), Ampicillin (AMP), Penicillin (P), Cefotaxime (CTX), Imipenem (IPM), Meropenem (MEM), Ciprofloxacin (CIP), Levofloxacin (LEV), Moxifloxacin (MXF), Gentamicin (CN), Vancomycin (VA), Tetracycline (TE), Erythromycin (E), Azithromycin (AZM), Clindamycin (DA), Linezolid (LZD), Metronidazole (MTZ), Rifampicin (RD), Trimethoprim-Sulfamethoxazole (SXT), Daptomycin (DAP), Fosfomycin (FOS), Nitrofurantoin (F).

## Data Availability

The data presented in this study are available in the main text.

## Supplementary Materials

The following supporting information can be downloaded at: https://www.mdpi.com/article/10.3390/jcm11051348/s1, Table S1: Articles in the systematic review of emerging microorganisms in the genitourinary system, Table S2: Most frequent emerging microorganisms in cases of suspected urinary infection by sex, Table S3: Most frequent emerging microorganisms in cases of suspected urinary infection by age, Table S4: Most frequent emerging microorganisms in genital samples by sex.
