# Supplementary material for: Emerging Presence of Culturable Microorganisms in Clinical Samples of the Genitourinary System: Systematic Review and Experience in Specialized Care of a Regional Hospital
Source: J Clin Med. 2022 Mar 1;11(5):1348. doi: 10.3390/jcm11051348 (PMC8911399; doi:10.3390/jcm11051348)
Supplement: Supplementary file 1 [file jcm-11-01348-s001.zip › jcm-1591230-supplementary.pdf]

Supplementary tables

Tabla S1: Articles in the systematic review of emerging microorganisms in the genitourinary system

1. Vedel G, Toussaint G, Riegel P, Fouilladieu J-L, Billöet A, Poyart C. Corynebacterium pseudogenitalium urinary tract infection. *Emerg Infect Dis*. 2006;12(2):355-356. doi:10.3201/eid1202.050950.
2. El Sayegh H, Elouardani M, Iken A, et al. [Encrusted cystitis due to Corynebacterium urealyticum]. *La Rev Med interne*. 2008;29(4):328-330. doi:10.1016/j.revmed.2007.10.405.
3. Pierciaccante A, Pompeo ME, Fabi F, Venditti M. Successful treatment of Corynebacterium urealyticum encrusted cystitis: a case report and literature review. *Le Infez Med*. 2007;15(1):56-58.
4. Beteta López A, Gil Ruiz MT, Vega Prado L, Fajardo Olivares M. [Cystitis and haematuria due to Corynebacterium striatum. A case report and review]. *Actas Urol Esp*. 2009;33(8):909-912. doi:10.1016/s0210-4806(09)72880-3.
5. Larios OE, Bernard KA, Manickam K, Ng B, Alfa M, Ronald A. First report of Actinobaculum schaalii urinary tract infection in North America. *Diagn Microbiol Infect Dis*. 2010;67(3):282-285. doi:10.1016/j.diagmicrobio.2010.02.023.
6. Zimmermann P, Berlinger L, Liniger B, Grunt S, Agyeman P, Ritz N. Actinobaculum schaalii an emerging pediatric pathogen? *BMC Infect Dis*. 2012;12:201. doi:10.1186/1471-2334-12-201.
7. Zelyas N, Gee S, Nilsson B, Bennett T, Rennie R. Infections Caused by Actinomyces neuii: A Case Series and Review of an Unusual Bacterium. *Can J Infect Dis Med Microbiol = J Can des Mal Infect la Microbiol medicale*. 2016;2016:6017605. doi:10.1155/2016/6017605
8. Barberis CM, Montalvo E, Imas S, et al. Total nephrectomy following Corynebacterium coyleae urinary tract infection. *JMM case reports*. 2018;5(9):e005149. doi:10.1099/jmmcr.0.005149
9. Jiménez-Guerra G, Lara-Oya A, Martínez-Egea I, Navarro-Marí JM, Gutiérrez-Fernández J. Urinary tract infection by aerococcus sanguinicola. An emerging opportunistic pathogen. *Rev Clin Esp*. 2018;218(7):351-355. doi:10.1016/j.rce.2018.04.002
10. Lorenzin G, Piccinelli G, Carlassara L, et al. Myroides odoratimimus urinary tract infection in an immunocompromised patient: an emerging multidrug-resistant micro-organism. *Antimicrob Resist Infect Control*. 2018;7:96. doi:10.1186/s13756-018-0391-4
11. Figueroa Rodriguez F, Faieta Lasarcina A, Davila Grijalva F. Mitral Valve Endocarditis with Perforation from a Urinary Source: An Unusual Case and Literature Review. *Case reports Cardiol*. 2019;2019:5496851. doi:10.1155/2019/5496851
12. Pichon M, Micaelo M, Longuet P, et al. A rare case of Corynebacterium riegelii urosepsis: Role of the MALDI-TOF mass spectrometry in the identification of emerging pathogens. *Med Mal Infect*. 2019;49(6):474-477. doi:10.1016/j.medmal.2019.06.005
13. Napolitani M, Troiano G, Bedogni C, Messina G, Nante N. Kocuria kristinae: an emerging pathogen in medical practice. *J Med Microbiol*. 2019;68(11):1596-1603. doi:10.1099/jmm.0.001023
14. Pollett S, Rocha C, Zerpa R, et al. Campylobacter antimicrobial resistance in Peru: a ten-year observational study. *BMC Infect Dis*. 2012;12:193. doi:10.1186/1471-2334-12-193
15. Karolus JJ, Gandelman AL, Nolan BA. Urethritis caused by Neisseria meningitidis. *J*

Clin Microbiol. 1980;12(2):284-285. doi:10.1128/jcm.12.2.284-285.1980

16. Chowdhury MN, Pareek SS. Urethritis caused by group B streptococci: a case report. *Br J Vener Dis*. 1984;60(1):56-57. doi:10.1136/sti.60.1.56
17. Noble RC. Colonisation of the urethra with Streptococcus pneumoniae: a case report. *Genitourin Med*. 1985;61(5):345-346. doi:10.1136/sti.61.5.345
18. Hay PE, Murphy SM, Chinn RJ. Acute urethritis due to Neisseria meningitidis group A acquired by oro-genital contact: case report. *Genitourin Med*. 1989;65(4):285-286. doi:10.1136/sti.65.4.285-a
19. Wilson AP, Wolff J, Atia W. Acute urethritis due to Neisseria meningitidis group A acquired by orogenital contact: case report. *Genitourin Med*. 1989;65(2):122-123. doi:10.1136/sti.65.2.122
20. Phillips EA, Shultz TR, Tapsall JW, Chambers IW. Maltose-negative Neisseria meningitidis isolated from a case of male urethritis. *J Clin Microbiol*. 1989;27(12):2851-2852. doi:10.1128/jcm.27.12.2851-2852.1989
21. Shanmugaratnam K, Pattman RS. Acute urethritis due to Neisseria meningitidis. *Genitourin Med*. 1989;65(6):401-402. doi:10.1136/sti.65.6.401-b
22. Faigel HC. Meningococcal urethritis. *J Adolesc Heal care Off Publ Soc Adolesc Med*. 1990;11(4):355-357. doi:10.1016/0197-0070(90)90048-7
23. Coker DM, Griffiths LR. Moraxella urethritis mimicking gonorrhoea. *Genitourin Med*. 1991;67(2):173-174. doi:10.1136/sti.67.2.173-a
24. Quarto M, Barbuti S, Germinario C, Vena GA, Foti C. Urethritis caused by neisseria meningitidis: a case report. *Eur J Epidemiol*. 1991;7(6):699-701. doi:10.1007/BF00218686
25. Kanemitsu N, Hayashi I, Satoh N, et al. Acute urethritis caused by Neisseria meningitidis. *Int J Urol Off J Japanese Urol Assoc*. 2003;10(6):346-347. doi:10.1046/j.1442-2042.2003.00621.x
26. Orden B, Martínez-Ruíz R, González-Manjavacas C, Mombiela T, Millán R. Meningococcal urethritis in a heterosexual man. *Eur J Clin Microbiol Infect Dis Off Publ Eur Soc Clin Microbiol*. 2004;23(8):646-647. doi:10.1007/s10096-004-1178-5
27. Cobo F, Jiménez G, Rodríguez-Granger J, Sampedro A. Posttraumatic Skin and Soft-Tissue Infection due to Pseudomonas fulva. *Case Rep Infect Dis*. 2016;2016:8716068. doi:10.1155/2016/8716068
28. Rodriguez CN, Rodriguez-Morales AJ, Garcia A, et al. Quinolone and azithromycin-resistant Neisseria meningitidis serogroup C causing urethritis in a heterosexual man. *Int J STD AIDS*. 2005;16(9):649-650. doi:10.1258/0956462054944363
29. Urra E, Alkorta M, Sota M, et al. Orogenital transmission of Neisseria meningitidis serogroup C confirmed by genotyping techniques. *Eur J Clin Microbiol Infect Dis Off Publ Eur Soc Clin Microbiol*. 2005;24(1):51-53. doi:10.1007/s10096-004-1257-7
30. Abdolrasouli A, Roushan A. Corynebacterium propinquum associated with acute, nongonococcal urethritis. *Sex Transm Dis*. 2013;40(10):829-831. doi:10.1097/OLQ.0000000000000027
31. Abdolrasouli A, Amin A, Baharsefat M, Roushan A, Hemmati Y. Moraxella catarrhalis associated with acute urethritis imitating gonorrhoea acquired by oral-genital contact. *Int J STD AIDS*. 2007;18(8):579-580. doi:10.1258/095646207781439775
32. Koroglu M, Yakupogullari Y, Aydogan F. A case of urethritis due to Streptococcus pneumoniae. *Sex Transm Dis*. 2007;34(12):1040. doi:10.1097/OLQ.0b013e31815b0168
33. Galan-Sanchez F, Aznar-Marin P, Marin-Casanova P, Garcia-Martos P, Rodriguez-Iglesias M. Urethritis due to Corynebacterium glucuronolyticum. *J Infect Chemother Off J Japan Soc Chemother*. 2011;17(5):720-721. doi:10.1007/s10156-011-0237-y
34. Katz AR, Chasnoff R, Komeya A, Lee MVC. Neisseria meningitidis urethritis: a case report highlighting clinical similarities to and epidemiological differences from

gonococcal urethritis. *Sex Transm Dis*. 2011;38(5):439-441. doi:10.1097/OLQ.0b013e3181ffa7dc

35. Bousquet A, Janvier F, Abi R, Larréché S, Mérens A. [Neisseria meningitidis urethritis]. *Med Mal Infect*. 2012;42(9):444-445. doi:10.1016/j.medmal.2012.07.016
36. Babics A, Roussellier P. Gardnerella vaginalis: An overlooked pathogen in male patients? *Med Mal Infect*. 2015;45(10):423-424. doi:10.1016/j.medmal.2015.09.007
37. Gherardi G, Di Bonaventura G, Pompilio A, Savini V. Corynebacterium glucuronolyticum causing genitourinary tract infection: Case report and review of the literature. *IDCases*. 2015;2(2):56-58. doi:10.1016/j.idcr.2015.03.001
38. Lo S, Thiam I, Fall B, et al. Urinary tract infection with Corynebacterium aurimucosum after urethroplasty stricture of the urethra: a case report. *J Med Case Rep*. 2015;9:156. doi:10.1186/s13256-015-0638-0
39. Grandolfo M, Vestita M, Bonamonte D, Filoni A. Acute Urethritis and Balonoposthitis Associated to Neisseria elongata. *Sex Transm Dis*. 2016;43(12):778-779. doi:10.1097/OLQ.0000000000000532.
40. Jannic A, Mammeri H, Larcher L, et al. Orogenital Transmission of Neisseria meningitidis Causing Acute Urethritis in Men Who Have Sex with Men. *Emerg Infect Dis*. 2019;25(1):175-176. doi:10.3201/eid2501.171102
41. Jaffe LR, Stavis JA. Isolation of Neisseria meningitidis from anogenital sites in adolescents: clinical implications. *J Adolesc Heal care Off Publ Soc Adolesc Med*. 1983;4(3):171-173. doi:10.1016/s0197-0070(83)80371-4
42. Quentin R, Fignon A, Lansac J, Goudeau A. Pasteurella multocida and female genital carcinoma; a case report to dispute the concept of genital opportunistic pathogen. *Eur J Obstet Gynecol Reprod Biol*. 1991;41(3):243-244. doi:10.1016/0028-2243(91)90031-f
43. Harriau P, Ramanantsoa C, Pierre F, Riou JY, Quentin R. Endocervical infection in a pregnant woman caused by Neisseria meningitidis: evidence of associated oropharyngeal colonization of the male partner. *Eur J Obstet Gynecol Reprod Biol*. 1997;74(2):145-147. doi:10.1016/s0301-2115(97)00098-5
44. Greif Z, Moscona M, Loeb D, Spira H. Puerperal Pasteurella multocida septicemia. *Eur J Clin Microbiol*. 1986;5(6):657-658. doi:10.1007/BF02013292
45. Vilà de Muga M, Pineda Solas V, Loverdos Eseverri I, Pérez Sánchez J, San Vicente Vela B, Argemí Renom S. [Streptococcus pneumoniae-induced recurrent vaginitis and peritonitis in a prepubertal child]. *An Pediatr (Barc)*. 2008;68(6):627-628. doi:10.1157/13123301
46. Chen X, Zhao X, Chen L, Zeng W, Xu H. Vaginitis Caused by Corynebacterium amycolatum in a Prepubescent Girl. *J Pediatr Adolesc Gynecol*. 2015;28(6):e165-7. doi:10.1016/j.jpag.2015.03.008
47. Gómez-Camarasa C, Fernández-Parra J, Navarro-Marí JM, Gutiérrez-Fernández J. [Moraxella osloensis emerging infection. Visiting to genital infection]. *Rev Esp Quimioter Publ Of la Soc Esp Quimioter*. 2018;31(2):178-181.
48. Tanpowpong P, Charoenmuang R, Apiwattanakul N. First pediatric case of Chromobacterium haemolyticum causing proctocolitis. *Pediatr Int*. 2014;56(4):615-617. doi:10.1111/ped.12301
49. Jiménez-Guerra G, Heras-Cañas V, Béjar Molina L del C, Sorlózano-Puerto A, Navarro-Marí JM, Gutiérrez-Fernández J. Extended-spectrum beta-lactamase-producing Escherichia coli and Klebsiella pneumoniae from urinary tract infections: Evolution of antimicrobial resistance and treatment options. *Med Clin (Barc)*. 2018;150(7):262-265. doi:10.1016/j.medcli.2017.07.023
50. Gutierrez-Fernandez J, Medina V, Hidalgo-Tenorio C, Abad R. Two Cases of Neisseria meningitidis Proctitis in HIV-Positive Men Who Have Sex with Men. *Emerg Infect Dis*. 2017;23(3):542-543. doi:10.3201/eid2303.161039

51. Nguyen C, Dascal A, Mendelson J. Prostatic abscess caused by Streptococcus mutans. *Can J Infect Dis = J Can des Mal Infect*. 1990;1(3):82-84. doi:10.1155/1990/797838
52. Qu L, Strollo DC, Bond G, Kusne S. Nocardia prostatitis in a small intestine transplant recipient. *Transpl Infect Dis*. 2003;5(2):94-97. doi:10.1034/j.1399-3062.2003.00022.x
53. Martinaud C, Gaillard T, Maslin J, et al. [Actinobaculum schaali bacteremia in an aged male patient]. *Med Mal Infect*. 2008;38(11):617-619. doi:10.1016/j.medmal.2008.09.013
54. Torres-Sangiao E, Lissarrague-Sanz A, Cañizares-Castellanos A, Bou G. [Bacterial prostatitis]. *Enferm Infecc Microbiol Clin*. 2013;31(5):344-346. doi:10.1016/j.eimc.2012.09.020
55. Siller Ruiz M, Hernández Egido S, Calvo Sánchez N, Muñoz Bellido JL. Unusual clinical presentations of Actinotignum (Actinobaculum) schaalii infection. *Enferm Infecc Microbiol Clin*. 2017;35(3):197-198. doi:10.1016/j.eimc.2016.09.006
56. Kawahara K, Mukai T, Miyaji Y, Morita Y. Chronic reactive arthritis associated with prostatitis caused by Neisseria meningitidis. *BMJ Case Rep*. 2018;2018. doi:10.1136/bcr-2017-223537

Tabla S2. Most frequent emerging microorganisms in cases of suspected urinary infection by sex.

MICROORGANISM	MALE N=59 (32,4%)	FEMALE N=123 (67,6%)	TOTAL
Streptococcus group bovis	19 (32,3%)	72 (58,5%)	91
Gardnerella vaginalis	9 (15,3%)	29 (23,6%)	38
Aerococcus urinae	11 (18,6%)	7 (5,7%)	18
Lactobacillus gasserii* y *L. delbrueckii	4 (6,8%)	9 (7,3%)	13
Corynebacterium glucuronolyticum* y *C. urealyticum	10 (16,9%)	2 (1,6%)	12
Aerococcus sanguinicola	6 (10,2%)	4 (3,3%)	10

Tabla S3. Most frequent emerging microorganisms in cases of suspected urinary infection by age.

MICROORGANISM	CHILDREN N=18 (9.9%)	ADULTS N=164 (90.1%)	TOTAL
Streptococcus group bovis	16 (88,9%)	75 (45,7%)	91
Gardnerella vaginalis	0 (0%)	38 (23,2%)	38
Aerococcus urinae	1 (5,6 %)	17 (10,4%)	18
Aerococcus sanguinicola	1 (5,6%)	9 (5,5%)	10
Lactobacillus gasserii* y *L. delbrueckii	0 (0%)	13 (7,9%)	13
Corynebacterium glucuronolyticum* y *C. urealyticum	0 (0%)	12 (7,3%)	12

Tabla S4. Most frequent emerging microorganisms in genital samples by sex.

MICROORGANISM	MALE N=59 (84,3%)	FEMALE N=11 (15,7%)	TOTAL
Gardnerella vaginalis	21 (35,6%)	0 (0%)	21
Corynebacterium glucuronolyticum	19 (32,2%)	0 (0%)	19

S.* group *viridans	3 (5,1%)	5 (45,5%)	8
Actinotignum schaalii	4 (6,8%)	1 (9,1%)	5
Facklamia hominis	4 (6,8%)	1 (9,1%)	5
Aerococcus urinae	3 (5,1%)	0 (0%)	3
Eikenella corrodens	0 (0%)	3 (27,3%)	3
Pasteurella bettyae	2 (3,4%)	1 (9,1%)	3
Streptococcus* group *bovis	3 (5,1%)	0 (0%)	3
